# The Rotterdam Elderly Pain Observation Scale (REPOS) is reliable and valid for non-communicative end-of-life patients

**DOI:** 10.1186/s12904-018-0280-x

**Published:** 2018-02-21

**Authors:** Anniek D. Masman, Monique van Dijk, Joost van Rosmalen, Frans P. M. Baar, Dick Tibboel, Anneke A. Boerlage

**Affiliations:** 1 Pain Expertise Centre, Erasmus MC-Sophia Children’s Hospital, P.O. Box: Postbus 2060, 3000 CA Rotterdam, The Netherlands; 2 Palliative Care Centre, Laurens Cadenza, Oosterhagen 239, 3078 CL Rotterdam, The Netherlands; 3 Intensive Care, Department of Pediatric Surgery, Erasmus MC-Sophia Children’s Hospital, P.O. Box: Postbus 2060, 3000 CA Rotterdam, The Netherlands; 4 Department of Biostatistics, Erasmus MC, P.O. Box: Postbus 2060, 3000 CA Rotterdam, The Netherlands

**Keywords:** pain measurement, palliative care, terminal care, hospice care, delirium, dementia, amnestic, cognitive disorders, consciousness disorders

## Abstract

**Background:**

In palliative care, administration of opioids is often indispensable for pain treatment. Pain assessment may help recognize pain and guide treatment in non-communicative patients. In the Netherlands the Rotterdam Elderly Pain Observation Scale (REPOS) is recommended to this aim, but not yet validated. Therefore the objective of this study was to validate the REPOS in non-communicative or unconscious end-of-life patients.

**Methods:**

In this observational study, the primary researcher applied the REPOS, while both the researcher and a nurse applied the Numeric Rating Scale (NRS). If possible, the patient in question applied the NRS as well. The NRS scores were compared with the REPOS scores to determine concurrent validity. REPOS scores obtained before and after a pain-reducing intervention were analysed to establish the scale’s sensitivity to change.

**Results:**

A total of 183 REPOS observations in 100 patients were analysed. Almost 90% of patients had an advanced malignancy; observations were done a median of 3 days (IQR 1 to 13) before death. Internal consistency of the REPOS was 0.73. The Pearson product moment correlation coefficient ranged from 0.64 to 0.80 between REPOS and NRS scores. REPOS scores declined with median 2 points (IQR 1 to 4) after a pain-reducing intervention (*p* < 0.001). Optimal sensitivity (0.81) and specificity (0.62) were found at cut-off score 3.

**Conclusions:**

This study demonstrates that the REPOS has promising psychometric properties for pain assessment in non-communicative end-of-life patients. Its application may be of additional value to relieve suffering, including pain, in palliative care.

**Electronic supplementary material:**

The online version of this article (10.1186/s12904-018-0280-x) contains supplementary material, which is available to authorized users.

## Background

Several studies reported that 45% to 70% of patients with incurable cancer, either admitted to a hospice or staying elsewhere, suffer moderate to severe pain [1–3]. Forty-five per cent of those patients receive opioids, which are the strongest analgesics [2, 4]. Pain levels should be accurately assessed to guide pain treatment.

Patients’ self-report of pain is considered the “gold standard” for pain assessment [5]. However, in the terminal phase of life, patients may not be able to self-report pain. To illustrate this, 68% to 83% of patients had cognitive failure [6, 7] and 90% to 98% of patients were drowsy or unresponsive prior to death [8–10]. Proxy pain assessment by a nurse was needed, therefore, in 90% of patients in a palliative care unit at the day of death [9]. Assessment of suffering, including pain, is challenging anyway in the terminal phase, especially in sedated patients [11].

Proxy assessment often underestimates the pain level, with consequent risk of under treatment [12, 13]. The risk might be lower if attention is paid to well-defined behaviour indicative for pain. Therefore, application of an observation scale that includes such behaviours (and has been validated for the target group) could be more beneficial for non-communicative patients.

We previously developed the Rotterdam Elderly Pain Observation Scale (REPOS, see Additional files 1, 2 and 3) in a study among nursing home residents with speech limitations caused by various disorders. The REPOS is available in Dutch and English [14, 15]. Dutch palliative guidelines recommended the REPOS for specific non-communicative patient groups [16], such as persons with dementia and intellectual disability. It proved a valid tool to measure pain in the nursing home population, including those who could not communicate [14]. However, the REPOS was not yet validated for palliative patients and needed to be re-validated for this population.

Beside the REPOS several other observational pain assessment tools have been developed for specific non-communicative patients groups [17–19], but to our best of knowledge only one of these has been validated for end-of-life patients, the Multidimensional Objective Pain Assessment Tool (MOPAT) [20]. This tool, published in 2011, was developed for hospice patients who are unable to self-report pain. It was tested in a small sample of 28 alert patients and 30 non-communicative patients and showed good internal consistency and sensitivity to change after a pain-reducing intervention [20]. A disadvantage of the MOPAT, however, is that it includes blood pressure and heart rate measurements, which are often stopped at the end of life, as recommended in the Liverpool Care Pathway for the dying patients [21].

In the Netherlands, the REPOS is increasingly adopted in nursing homes and institutions for intellectually disabled or non-communicative patients [15]. Hospice patients, however, may have other characteristics. They often suffer from advanced cancer (> 90% of patients) and are mostly bedridden. Self-report is not possible due to their illness state (comatose, delirium or adverse effects of medication), in contrast to nursing home patients who more often have dementia. In addition, end-of-life patients are in another emotional state and may be extremely anxious, facing death. All these aspects may influence experiences or expressions of pain [17].

## Methods

### Aim

The aim of this study was to revalidate the REPOS for pain assessment in non-communicative or unresponsive end-of-life patients.

### Design, participants and setting

This observational study was performed in Laurens Cadenza in Rotterdam. This is the largest palliative care centre in the Netherlands, with 20 beds for end-of-life care and symptom management; 200 to 250 patients are admitted annually. The main admission criterion is having a life expectancy of less than 3 months. Approximately 90% of the patients have advanced malignant disease. The median length of stay in 2010 was 11 days (IQR 5 to 29) and the discharge rate was 4% [22]. Dutch palliative guidelines are adhered to [16]. A multidisciplinary team of health care professionals, including caregivers, nurses and elderly care physicians specialized in palliative care, is available 24 h per day. In addition, many volunteers provide support. Despite a small difference in duration of admission the included patients can be seen as representative of all patients admitted to Cadenza during the year 2010 [22].

### Assessment tools

The Rotterdam Elderly Pain Observations Scale (REPOS) consists of 10 behavioural items (see Additional files 1, 2 and 3), which the observer scores as present or absent after having observed the patient for two minutes preferably during a possible painful moment of care [14]. To optimize inter-observer reliability, a definition chart, which describes all 10 items in detail, and an intervention decision tree are provided (see Additional files 1, 2 and 3). To ascertain sufficient interrater reliability of a REPOS observation, nurses receive training including about 10 bedside paired observations with an experienced REPOS observer [23, 24]. Sufficient interrater reliability is defined as Cohen’s kappa> 0.65. A previous validation study in nursing home residents revealed a significant difference between painful and rest situations and a large correlation with the PAINAD (r = .75) indicating good construct validity. For nursing home residents both the sensitivity (.85) and the specificity (.83) were optimal at a cut-off score of 3 [14]. However, as behaviour might be the result of other emotions than pain, the observer in addition estimates the pain intensity on a Numeric Rating Scale (NRS) from 0 (no pain) to 10 (worst possible pain). Thus, assigning an ‘NRS-observer’ score is a standard part of the REPOS observation [15]. A REPOS score of 3 or higher in combination with a NRS-observer score of 4 or higher suggests moderate to severe pain for which an intervention is required [14, 25]. The NRS is considered a valid tool to assess cancer pain intensity [26, 27].

### Procedure

Data were collected during three phases, based on the implementation of REPOS and its use in standard care in Laurens Cadenza: training, application in daily practice, and sensitivity-to-change data collection.

First, from March to October 2010, the primary investigator (A.M.) trained nurses in Laurens Cadenza to assess pain with the REPOS, since at that time symptom measurement was not a standard of care. Firstly, the primary investigator performed 10 bedside observations simultaneously with an experienced REPOS observer, to ascertain sufficient interrater reliability for her. As Cohen’s kappa for the primary investigator was established at 0.76, she was qualified to perform REPOS observations in this study. November–December 2010, NRS and REPOS assessments were implemented in daily practice for all non-communicative or unresponsive patients. All trained nurses achieved good interrater reliability with the primary investigator after 6 to 10 paired observations (Cohen’s kappa values ranged from 0.70 to 0.78).

Second, from January 2011 to May 2012, the primary investigator or a trained nurse assigned a REPOS score and an NRS-observer score in daily practice as standard of care.

Third, from February to June 2013, the second investigator (A.B.) was called in when a patient received a pain-reducing intervention and assigned a REPOS score just before and at least one hour after this intervention. These pre- and post-intervention data were used for the sensitivity-to-change analysis.

To determine internal consistency, concurrent validity and the optimal cut-off score, all REPOS observations made during the three different phases were included, with the exception that regarding the training phase only the observations by the primary investigator were selected and not those of the nurses in training. For the sensitivity-to-change analysis only the pre- and post-intervention data from phase three were used.

The type of pain assessment (REPOS observation or self-report) depended on the patient’s ability to communicate. In the case of non-communicative or unresponsive patients, the primary investigator assigned a REPOS score and a NRS (NRS-observer), and also the caregiving nurse assigned a NRS score (NRS-proxy). Communicative patients reported in addition to the REPOS score an NRS score themselves (NRS-patient).

### Other variables

Demographic characteristics (age, gender, diagnoses, and duration of admission) were extracted from the electronic medical records; the primary diagnoses and the number of comorbidities were evaluated. The primary diagnoses refer to the WHO’s International Classification of Diseases (ICD-10 classification) coding for the patient’s terminal illness.

Analgesics prescribed at the time of observation were retrieved from the patients’ medical file and classified according to the WHO three-step pain ladder as step 1 (non-opioids; acetaminophen and NSAIDs), step 2 (weak opioids) and step 3 (strong opioids) [28, 29]. The highest-step analgesic prescribed for a patient over all the observations is given in the results section under the heading patient characteristics.

### Data analysis

Data are presented as mean (standard deviation; SD) in case of normally distributed variables and as median (interquartile range = IQR or minimum-maximum range = range) in case of non-normally distributed variables.

To determine interrater reliability, Cohen’s kappa was applied for the primary and second investigator and for all nurses who assigned REPOS scores, and defined as good if ≥ 0.65 [30].

Cronbach’s alpha coefficient served to examine the internal consistency reliability of the REPOS items; a value of at least 0.70 is considered good reliability [31, 32]. Pearson product moment correlation coefficient was applied to determine concurrent validity of the REPOS with the NRS scores. This validity coefficient should exceed 0.30 [33]. The Wilcoxon signed rank test served to estimate sensitivity to change after a pain intervention. The optimal cut-off value for REPOS score was determined as the best combination of sensitivity and specificity comparing the REPOS total scores with NRS proxy as reference.

The correlations between the REPOS, NRS proxy and NRS observer in a repeated measurements setting were calculated with linear mixed modelling using the method proposed by Hamlett et al. [34]. In the linear mixed model, we adjusted the outcomes REPOS, NRS proxy and NRS observer for the independent variables gender, assessment number (repeated assessments per person) and time to death.

Data analyses were performed using IBM SPSS Statistics 24. A significance level of 0.05 (two-sided) was used for statistical tests.

## Results

### Patient characteristics

Over the three study phases, 194 REPOS scores were assigned to 103 patients. Data from three patients were excluded from analysis because these observations were not considered end-of-life assessments: one patient had been discharged after the observations were made and in two data had been obtained earlier than three months before death. Data of the remaining 100 patients were included in the analysis. For those included patients, the first (or only) observation was done a median of 3 days (IQR 1 to 13) before death. The median age was 77 years (IQR 67 to 85), 65% were female, and the median duration of admission was 28 days (IQR 9 to 51). Advanced malignancy, mainly of digestive and respiratory organs, was the main reason for admission (89% of patients). Most patients (73%) were receiving a standing dose of strong opioids combined with a rescue prescription for breakthrough pain. Six percent of all patients had an ‘as needed’ opioids prescription only, and 11% received no analgesics at all. Patient characteristics are shown in Table 1.

**Table 1:** Patient characteristics

Characteristics	*N* = 100
Gender in %	
Male / female	35 / 65
Age in years	
median (IQR)	77 (67 to 85)
Duration of admission in days	
Median (IQR)	28 (9 to 51)
Assessments days before death	
Median (IQR)	3 (1 to 13)
Primary diagnose in N (%)	
Neoplasms	89
*Digestive organs*	*26 (29)*
*Respiratory and intra-thoracic organs*	*17 (19)*
*Female genital organs*	*9 (10)*
*Breast*	*7 (8)*
*Eye, brain and other parts of central nervous system*	*7 (8)*
*Lymphoid, hematopoietic and related tissue*	*7 (8)*
*Ill –defined, secondary and unspecified sites*	*7 (8)*
*Other*	*9 (10)*
Disease of nervous system	4 (acquired brain injury; Parkinson’s disease; systemic atrophy)
Infectious and parasitic disease	3 (pneumonia and frailty)
Other	4 (CVA; lung disease, kidney failure, invalidity)
Analgesics in %	
Opioids around the clock	73
None	11
Non-opioids around the clock	9
Opioids as needed	6
Non-opioids as need	1

### REPOS scores and NRS scores

The number of REPOS observations for the 100 patients included in the analysis was 183. The REPOS has been applied once in 52 patients and twice or more (range between 2 and 13) in 46. The observations were conducted by the observer when caregivers provided care, i.e. 34% during washing or dressing, 30% during posture change, 21% during rest, 9% during a transfer, and 6% in other care situations.

**Fig. 1:**
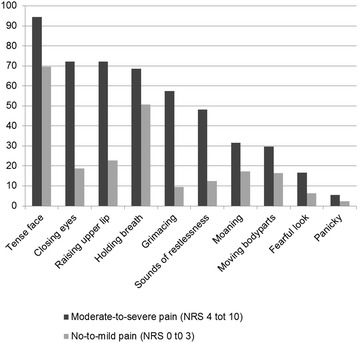
Percentage of scored REPOS items for no-mild pain and for moderate-to-severe pain

The median REPOS score was 3 (IQR 1 to 5); the median NRS-observer, NRS-proxy and NRS-patient scores were 2 (IQR 0 to 4), 3 (IQR 1 to 6) and 6 (IQR 2 to 7), respectively. REPOS scores indicative of pain (3 to 10) were assigned in 55% (101/183) of observations. Pain was rated moderate to severe (NRS 4 to 10) in 30% to 67% of NRS scores. All 10 REPOS items were scored as present more frequently in association with NRS scores 4 to 10 than in association with lower NRS scores (Table 2). The items ‘tense face’, ‘raising upper lip’ and ‘closing eyes’ were the ones most often observed in association with NRS scores of 4 to 10; the items ‘fearful look’ and ‘panicky’ the least often (Fig. 1).

**Table 2:** Pain assessments results

	Median score (IQR)	Moderate to severe pain (NRS 4 to 10) Number of observations (%)
REPOS score (*N* = 183)	3 (1 to 5)	101 (55)
NRS-observer (*N* = 182)	2 (0 to 4)	54 (30)
NRS-proxy (*N* = 107)	3 (1 to 6)	47 (44)
NRS-patient (*N* = 24)	6 (2 to 7)	16 (67)

### Internal consistency

The Cronbach’s alpha coefficient for internal consistency of the REPOS was 0.73. The item-total correlations ranged from 0.18 to 0.69, and were below 0.30 for items: panicky, fearful look, moaning and moving body parts.

### Concurrent validity

The REPOS was correlated to the NRS-observer, NRS-proxy and NRS-patient separately. The Pearson product moment correlation coefficient ranged from 0.64 (95% CI 0.51 to 0.74) to 0.80 (95% CI 0.72 to 0.86) (Table 3). Linear mixed modelling revealed a correlation between 0.64 and 0.78 for the REPOS, NRS proxy and NRS observer corrected for gender, assessment number (repeated assessments per person) and time to death.

**Table 3:** Correlation between REPOS score and the various NRS scores

		REPOS score	NRS-observer	NRS-proxy	NRS-patient
REPOS score	*Number of observations*	*183*	*182*	*107*	*24*
	R	–	0.73	0.64	0.66
	95% CI	–	0.65 to 0.79	0.51 to 0.74	0.35 to 0.84
NRS-observer	*Number of observations*		*182*	*107*	*24*
	R		–	0.80	0.77
	95% CI		–	0.72 to 0.86	0.53 to 0.90
NRS-proxy	*Number of observations*			*107*	*24*
	R			–	0.72
	95% CI			–	0.45 to 0.87
NRS-patient	*Number of observations*				*24*
	R				–
	95% CI				–

*Note*. *Abbreviations*: *REPOS* Rotterdam Elderly Pain Observation Scale, *NRS* Numeric Rating Scale, *R* Pearson correlation, *Cl* Confidence Interval

Deleting the above-mentioned 4 items with low correlations for internal consistency had hardly any effect on the Pearson coefficients, which then ranged from 0.62 (95% CI 0.49 to 0.73) to 0.80 (95% CI 0.72 to 0.86).

### Sensitivity to change

Twenty-two pairs of before-and-after scores were included for the sensitivity-to-change analysis. Twenty-one concerned a pharmacological pain-reducing intervention; the other pair concerned changing the patient’s posture to relieve or prevent pressure sores.

The median REPOS score declined after a pain-reducing intervention, both pharmacological and non-pharmacological, from 4 (IQR 3 to 6) to 1 (IQR 1 to 3) with a median reduction of 2 points (IQR 1 to 3). This change was statistically significant (*p* < 0.001). The percentage of REPOS scores indicating no pain (score 0 to 2) increased from 9% (2/22) to 68% (15/22).

### Cut-off score

**Fig. 2:**
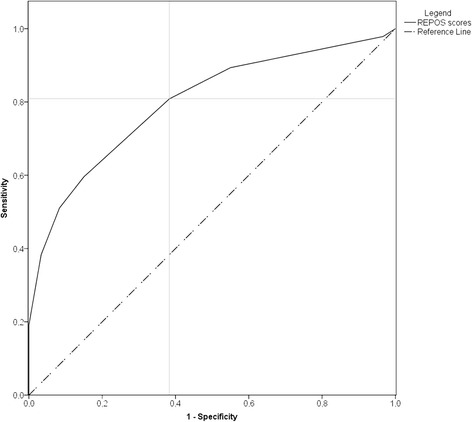
ROC curve of the optimal sensitivity and specificity for the REPOS score. The full line represents sensitivity and specificity of de REPOS score. The dotted line represents the line for which sensitivity and specificity are equal. A horizontal and a vertical grey line are added to show the optimal cut-off value of the REPOS score

In 107 observations, both a REPOS score and a NRS-proxy score were available. At the cut-off REPOS score of 3, sensitivity was 0.81 and specificity was 0.62. The ROC curve, with an AUC of 0.80 (95% CI 0.71 to 0.88), is displayed in Fig. 2. The positive predictive value was 0.62 and the negative predictive value was 0.80.

In 21% (23/107) of observations the REPOS score was indicative for pain (score 3–10) whereas the NRS-proxy score was not; suggesting false-positive outcomes of the REPOS. In contrast, in 8% (9/107) of observations the REPOS score did not indicate pain (score 0–2) whereas the NRS-proxy score did (score 4–10), suggesting false-negative outcomes.

## Discussion

The findings from this study show that the REPOS has promising psychometric properties to assess pain in non-communicative end-of-life patients; including adequate internal consistency, sufficient concurrent validity and good sensitivity to change after a pain-reducing intervention.

A variety of observational pain scales have been developed for other settings where non-communicative patients are treated, including intensive care units [35] and nursing homes [19]. Only one, the MOPAT [20], has been validated for non-communicative end-of-life patients, albeit preliminary and without establishing a cut-off score. The MOPAT was published after start of our study (2010). We therefore could not use it, although it would have been interesting to compare the two scales.

The overall internal consistency of the REPOS in the present study was adequate as judged from the Cronbach’s alpha coefficient of 0.73 [31, 32]. Although the item-total correlations of 4 items (panicky, fearful look, moaning and moving body parts) were below 0.30, we chose to retain those items. These low values could be related to infrequent occurrence of those behaviours in terminally ill patients as they receive high doses of strong analgesics and/or sedatives. For example, the ability to fully react with all body parts is often diminished in the end-of-life stage. In other non-communicative patients who use lower doses analgesics those behaviours might be more obvious. In observations of nursing home residents during a possible painful situation, i.e. washing and clothing or during physiotherapy, when manipulation irritates already affected tissue (i.e. arthritis) all 10 behaviours were seen. Deletion of those 4 items could therefore create a risk of underestimating pain in a broader group of non-communicative patients. The major reason for retaining these items is that the scale should be applicable in other settings with non-communicative patients as well.

With respect to concurrent validity, a high correlation (0.73) was found – not surprisingly – between REPOS and NRS scores assigned by the same person. The correlation between REPOS and the gold standard (NRS-patient) was only moderate [32], as is seen in other pain scale studies too [36, 37]. This moderate correlation is perhaps explained by patient characteristics. Patients who are unable to report pain with the NRS seem to show more nonverbal behaviour, such as grimace [36, 38]. For these patients their pain would be better reflected by a behavioural score than by a proxy NRS only.

It is not unexpected that the REPOS score and the NRS-proxy score may differ to some extent. A high REPOS score combined with a lower NRS-proxy score, or a so-called false positive score, is typically seen in patients who show “emotional” behaviour not related to pain, but based on anger, fear or agitation [39, 40]. The opposite, a false-negative score, may occur when the attending nurse has observed behaviour not included in the REPOS score, such as muscle tension. Alternatively, the nurse’s NRS score reflects knowledge of relevant characteristics, such as history or medication use, illness and other patient specific characteristics [12, 13, 41].

This study showed that the cut-off score of 3 or higher is applicable for non-communicative end-of-life patients. However, application of a ‘one-fits-all’ cut-off score is debated [42, 43]. A reason suggested in literature is that different underlying conditions cause different types of damage to the brain, and consequently different responsiveness to pain [43–45]. Based on these arguments one could consider the use of an individualized cut-off score, which has been recommended for other vulnerable patients groups, e.g. young children [46]. However, this approach asks more from the caregivers: it is a dynamic approach requiring evaluation and adjustment at regular times and when indicated. As daily pain assessment itself was shown to be problematic [47], one can wonder if an individualized approach is feasible in a daily care situation [48, 49].

A strength of the present study is that most observations were done within the last two weeks of life and therefore including even those patients near the time of death. In the previous validation study of the REPOS [14], only a small proportion of the population was at the end of life. In addition, communicative patients rated their pain themselves, which enabled comparison between the REPOS and the gold standard of self-report. Also, the sample size in the present study was 100 patients, which far exceeds the minimal number of 50 patients [31, 50].

Some limitations of this study have to be addressed, however. First, this is a single-centre study in mainly advanced cancer patients and care must be exercised in extrapolating the findings to other settings and patients populations, like palliative home care or community-based palliative care. Nevertheless, it seems unlikely that pain behaviour would be different in a different palliative environment. Second, the sensitivity-to-change analysis concerned only a relatively small sample. This limitation is encountered in many other psychometric studies, seeing that researchers often are not available when patients receive additional analgesia and also because nurses may tend to forego reassessment after a pain-reducing intervention [51]. The comparable MOPAT study [20] also included fewer patients than planned, mainly due to logistical reasons. The fact that the observer knew whether the patient received pain medication or not, could be considered a weakness of the sensitivity-to-change analysis. In the ideal situation, observers blinded for this condition apply the REPOS when watching video recordings made before and after an intervention. However, it was felt undesirable to ask relatives’ approval to film their loved ones in the dying phase for research purposes. Still, knowing that the REPOS is sensitive enough to measure small changes after an intervention means that it is suitable for pharmacodynamic studies, which are urgently needed in this palliative patient group [11]. Lastly, in the present study the REPOS was compared with NRS scores, although a comparison with another behaviour pain scale would have strengthened the reliability and validity testing [52]. In future studies the outcomes of different observational scales like the PACSLAC, PAINAD and MOPAT should be compared and the possibility should be explored if blinded observation is possible.

## Conclusions

In conclusion, the REPOS seems to meet the criteria for the use of a pain measurement tool in palliative care of the Expert Working Group of the European Association of Palliative Care (EAPC) [53]. That is, the brevity of the scale (10 well-defined behaviors, scored yes/no after a two-minute observation period) and the cut-off score increases clinical utility. The scale was validated for nursing home residents with speech limitations and the present study increases the psychometric knowledge about sensitivity to treatment effect and reliability during palliative care. The fact that the REPOS is available in Dutch as well as English can be seen as an advantage as well. In addition, next to the Dutch palliative guidelines [16], the use of the REPOS for pain assessment in non-communicative patients is recommended in a report on quality indicators in palliative care, published by the Netherlands Institute for Health Services Research [54]. We have demonstrated that the REPOS seems a valid tool for the assessment of pain in non-communicative end-of-life patients. We recommend its use on a daily basis for every non-communicative palliative patient. After a brief training course every professional palliative caregiver will be able to use it in daily practice. A REPOS electronic educational module is available (both in Dutch or English) to guide implementation and training [15].

## Additional files

Additional file 1: Rotterdam Elderly Pain Observation Scale (REPOS) score form. (PDF 638 kb)

Additional file 2: Rotterdam Elderly Pain Observation Scale (REPOS) instruction chart. (PDF 962 kb)

Additional file 3: Rotterdam Elderly Pain Observation Scale (REPOS) decision tree. (PDF 787 kb)

## Abbreviations

EAPC: European Association of Palliative Care
IQR: Interquartile range
MOPAT: Multidimensional Objective Pain Assessment Tool
NRS: Numeric Rating Scale
NRS-observer: NRS score assigned by the same person who performed the observation for the REPOS score
NRS-patient: NRS score given by a patient himself
NRS-proxy: NRS score assigned by a caregiving nurse
PAINAD: Pain Assessment in Advanced Dementia
REPOS: Rotterdam Elderly Pain Observation Scale
SD: standard deviation
