# Supplementary material for: The Rotterdam Elderly Pain Observation Scale (REPOS) is reliable and valid for non-communicative end-of-life patients
Source: BMC Palliat Care. 2018 Feb 21;17:34. doi: 10.1186/s12904-018-0280-x (PMC5822491; doi:10.1186/s12904-018-0280-x)
Supplement: Supplementary file 1 — Rotterdam Elderly Pain Observation Scale (REPOS) score form. (PDF 638 kb) [file 12904_2018_280_MOESM1_ESM.pdf]

# Rotterdam Elderly Pain Observation Scale (REPOS)

Please observe for 2 minutes, and tick the box if the specific behavior was present during the observation. Next, summate all ticked behaviors to obtain the REPOS total score.

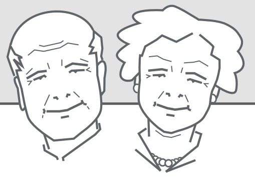

NAME CLIENT

| | 1st observation | 2nd observation | 3rd observation |
|---|---|---|---|
| NAME OBSERVER | | | |
| DATE/TIME | | | |
| SITUATION (ADL, transfer, walking, physical therapy, rest, wound care, e.g.) | | | |
| PAIN MEDICATION (type, dosing and time of last administration) | | | |
| Tense face | ☐ | ☐ | ☐ |
| Eyes (almost) squeezed | ☐ | ☐ | ☐ |
| Raising upper lip | ☐ | ☐ | ☐ |
| Grimace | ☐ | ☐ | ☐ |
| Frightened, fearful look | ☐ | ☐ | ☐ |
| Moving body parts | ☐ | ☐ | ☐ |
| Panicky, panics attack | ☐ | ☐ | ☐ |
| Moaning / groaning | ☐ | ☐ | ☐ |
| Sounds of restlessness / verbal expressions | ☐ | ☐ | ☐ |
| Breath holding / faltering respiration | ☐ | ☐ | ☐ |
| **REPOS TOTAL SCORE** | ☐ | ☐ | ☐ |
| | see REPOS decision tree | see REPOS decision tree | see REPOS decision tree |

REPOS© versie 1.2, Van Herk, Boerlage, Van Dijk, Erasmus MC 2008

Copyright 2009 Erasmus MC and Laurens, All rights reserved.
