# Supplementary material for: The Rotterdam Elderly Pain Observation Scale (REPOS) is reliable and valid for non-communicative end-of-life patients
Source: BMC Palliat Care. 2018 Feb 21;17:34. doi: 10.1186/s12904-018-0280-x (PMC5822491; doi:10.1186/s12904-018-0280-x)
Supplement: Supplementary file 2 — Rotterdam Elderly Pain Observation Scale (REPOS) instruction chart. (PDF 962 kb) [file 12904_2018_280_MOESM2_ESM.pdf]

Rotterdam Elderly Pain Observation Scale (REPOS)

Instruction chart

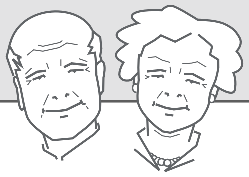

Definitions of the behaviors

Each item in the REPOS represents specific behavior or a certain reaction. The REPOS does not score intensity of behavior, but rather occurrence, yes or no. An item is only scored as present if the behavior in question was clearly visible. Scoring is not useful if the client is in relaxed sleep.

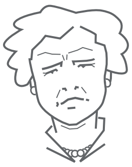

Tense face
One or more facial muscles are being tightened (are not relaxed).
This is NOT scored when client is talking.

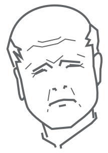

Eyes (almost) squeezed
Eyes tightly shut or squeezed.
Do NOT score if client shows blinking eyes or eyes shut without squeezing.

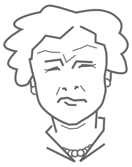

Raising upper lip
The upper lip is being pulled up, shortening the distance between upper lip and nose; nasio-labal furrows deepened, nostrils raised and enlarged.

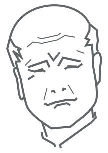

Grimace
Scored as present only when the following three facial expressions occur together:
1) Eyebrows drawn together and downward, with the skin fold between the eyebrows bulged out.
2) Eyes tightly shut or squeezed.
3) Nasio-labal furrows deeper than normal and drawn up sideways.

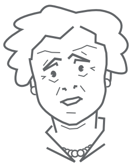

Frightened, fearful look
Large, widely opened eyes, and inner sides of eyebrows slightly raised and drawn together.

Moving body parts
Each movement indicative of resistance or protecting a (painful) body part. Included are movements such as changing one's position in a chair so as to relieve one's bottom, and grasping the head.
Do NOT score when the movement or action is functional, e.g. pushing one's hair out of one's face, or raising arms to take off clothes.

Panicky, panics attack
An extreme manifestation of anxiety showing in random nervous body movements or fierce resistance. This may co-occur with:
- *Frightened expression characterized by large, widely opened eyes, and inner sides of eyebrows slightly raised and drawn together; and/or*
- *Intense screams or verbal expressions of pain, such as 'ouch' or "you're hurting me'.*

Moaning/groaning
Monotonous and whining sound.

Sounds of restlessness/verbal expressions
Sudden or persisting intense screams or verbal expressions of pain, such as 'ouch' or "you're hurting me'.

Breath holding/faltering respiration
Briefly interrupted breathing, gasping.

Copyright 2009 Erasmus MC and Laurens, All rights reserved.
