# Supplementary material for: The Rotterdam Elderly Pain Observation Scale (REPOS) is reliable and valid for non-communicative end-of-life patients
Source: BMC Palliat Care. 2018 Feb 21;17:34. doi: 10.1186/s12904-018-0280-x (PMC5822491; doi:10.1186/s12904-018-0280-x)
Supplement: Supplementary file 3 — Rotterdam Elderly Pain Observation Scale (REPOS) decision tree. (PDF 787 kb) [file 12904_2018_280_MOESM3_ESM.pdf]

REPOS decision tree

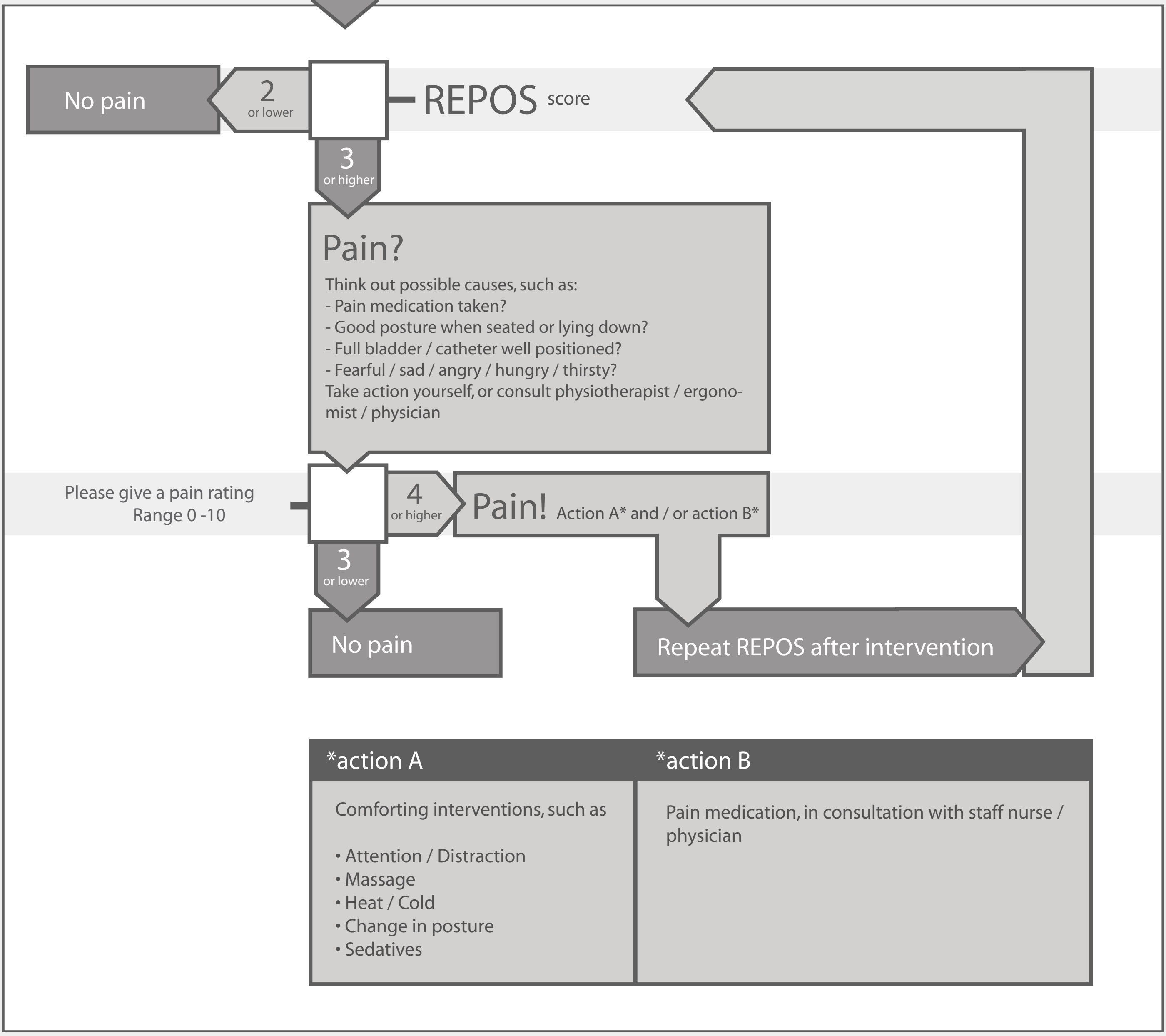

Please enter the REPOS score and pain rating in the schedule below, as well as the action taken.
The pain rating ranges from 0 (no pain) to 10 (worst imaginable pain).

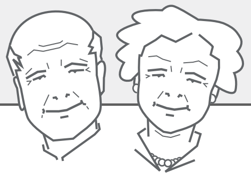

NAME CLIENT

	1st observation	2nd observation	3rd observation
NAME OBSERVER			
DATE/TIME			
REPOS score			
PAIN RATING			
ACTION			
